# Three-dimensional heterotypic colorectal cancer spheroid models for evaluation of drug response

**DOI:** 10.3389/fonc.2023.1148930

**Published:** 2023-07-04

**Authors:** Jia Ning Nicolette Yau, Giulia Adriani

**Affiliations:** ^1^Department of Pharmacy, Faculty of Science, National University of Singapore, Singapore, Singapore; ^2^Singapore Immunology Network (SIgN), Agency for Science, Technology and Research (A*STAR), Singapore, Singapore; ^3^Department of Biomedical Engineering, Faculty of Engineering, National University of Singapore, Singapore, Singapore

**Keywords:** spheroid, heterotypic 3D model, colorectal cancer, cancer associated fibroblast (CAF), endothelial cell, gut microbiota, drug screening, tumor associated macrophages (TAMs)

## Abstract

Colorectal cancer (CRC) is a leading cause of death worldwide. Improved preclinical tumor models are needed to make treatment screening clinically relevant and address disease mortality. Advancements in 3D cell culture have enabled a greater recapitulation of the architecture and heterogeneity of the tumor microenvironment (TME). This has enhanced their pathophysiological relevance and enabled more accurate predictions of tumor progression and drug response in patients. An increasing number of 3D CRC spheroid models include cell populations such as cancer-associated fibroblasts (CAFs), endothelial cells (ECs), immune cells, and gut bacteria to better mimic the *in vivo* regulation of signaling pathways. Furthermore, cell heterogeneity within the 3D spheroid models enables the identification of new therapeutic targets to develop alternative treatments and test TME-target therapies. In this mini review, we present the advances in mimicking tumor heterogeneity in 3D CRC spheroid models by incorporating CAFs, ECs, immune cells, and gut bacteria. We introduce how, in these models, the diverse cells influence chemoresistance and tumor progression of the CRC spheroids. We also highlight important parameters evaluated during drug screening in the CRC heterocellular spheroids.

## Introduction

Colorectal cancer (CRC) is the third most common cancer in males and the second most common cancer in females worldwide and continues to be a leading cause of death (1, 2). Reliable cancer models are imperative to advance cancer research and treatment (3). The traditional two-dimensional (2D) cell culture models have been critical in developing many first-line chemotherapeutics, such as cisplatin (4, 5). However, the limitations of 2D culture models prevent them from effectively recapitulating the physiological characteristics of native tumors. A key limitation of 2D cultures is the change in cell morphology, signaling, and functions compared to *in vivo* conditions in response to different external stimuli from the culture substrate and the neighboring cells (5–7). Consequently, 2D tumor models often overscore the effectiveness of potential drug candidates, resulting in lower efficacy and greater toxicity than predicted when translated into *in vivo* animal models or clinical trials (8). Patient-derived tumor xenograft and *in vivo* tumor models have been important for rational drug design and predicting response and side effects of chemotherapeutic regimens (9, 10). However, animal models often show a low success rate of engraftment (11), are expensive, require a cross-species comparison, and raise ethical controversies, challenging their utilization.

The TME is a complex and dynamic environment around the tumor composed of blood vessels, fibroblasts, immune cells, mesenchymal stromal cells, extracellular matrix, and cell-secreted factors (12). The TME is now recognized as a leading player in tumor development and response to chemo and immunotherapeutic strategies (12, 13). Therefore, recapitulating *in vitro* the heterogeneous human TME by introducing its main constituents in a three-dimensional (3D) format is essential for developing preclinical models with greater clinical relevance than 2D systems.

In this scenario, 3D tumor spheroid cultures that utilize hydrogels made of natural biomaterials (e.g. collagens, fibrin, hyaluronic acid) or synthetic polymers have been gaining increasing attention to better recapitulate the structure of tissues and native tumors compared to 2D cultures (3, 7, 14, 15). The development of 3D tumor spheroid cultures has prompted a paradigm shift in cancer research toward more clinically-relevant models, further fueled by advancements in biotechnologies. For instance, improvements in sampling and storage techniques allow clinicians to culture patient-derived 3D spheroids to identify genetic markers to predict disease progression and chemoresistance (16, 17). Tissue engineering techniques, such as the synthesis of scaffolds mimicking the extracellular matrix (ECM), and advances in microfluidic devices have improved the culture of spheroids in 3D settings to take into consideration cell-ECM and cell-cell interactions leading to a greater correspondence with native tumors compared to 2D cultures (18–24).

Specifically for CRC, recent reviews of 3D spheroid models highlight the utility of spheroids for drug screening (25), nanomedicine screening (26), and biomarker discovery (27). These reviews discuss strategies for adapting spheroids of various complexities for drug screening and developing better treatment strategies. However, these reviews only partially address the significance of recapitulating the heterogeneity of the CRC TME for drug screening.

Various 3D CRC spheroid models were derived from cancer cell lines only (monoculture) and used for drug screening (28–32) with success in modeling hypoxia and necrosis associated with tumor resistance to drugs (33).

**Figure 1:**
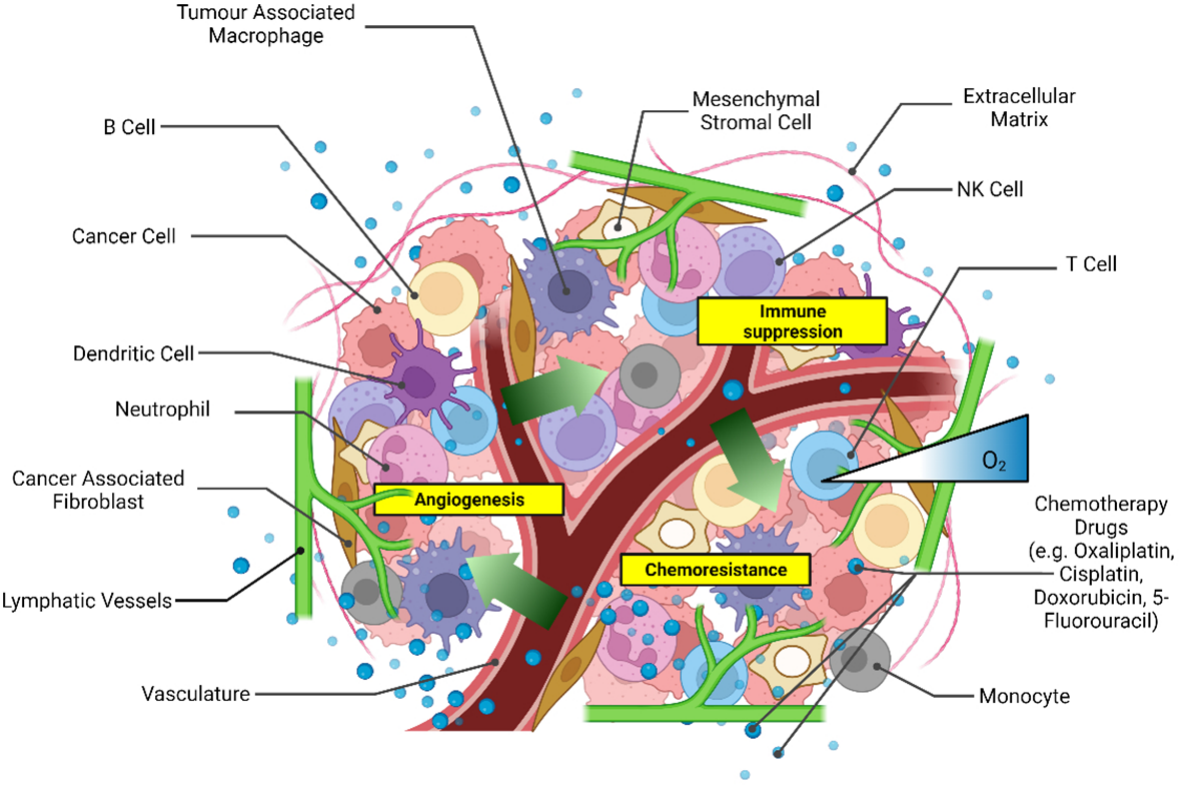
Scheme of the main components of the tumor microenvironment that could be recapitulated in heterotypic 3D CRC spheroid models for drug screening. Created with Biorender.com.

CRC patient-derived xenografts (34) and patient-derived cells (35, 36) have also been used for drug screening predicting the efficacies of chemotherapy regimens in personalized medicine, as extensively discussed in another review (37). Patient-derived spheroids enable the recapitulation of essential tumor tissue characteristics, such as the integrity of the genomic profile (38). A critical limitation of the patient-derived spheroid model remains the accessibility of the tissue and the success rate of spheroid formation. Unlike commercially available cell lines, *in vitro* cultures of patient-derived cells require skilled technical personnel for consistent cell isolation and culture conditions. Cell dissociation methods, either mechanical or enzymatic, can dramatically affect the yield and quality of the isolated cells (39). Conversely, commercially available cancer cell lines are ideal for reproducible high-yield production of 3D CRC spheroids for drug testing. 3D tumor spheroid models with an increased cellular complexity have been developed by culturing heterogeneous cell types within the spheroid, such as fibroblasts (40, 41), immune cells (42, 43), and endothelial cells (44, 45). These models aim to emulate the heterogeneity of the TME better to achieve a more significant physiological association with native tissue (**Figure 1**). Broadening the heterogeneity of 3D cultures is essential to drug development as cytokines released from immune cells and fibroblasts are known to modulate chemoresistance (41, 46). However, the validation of cell-line-derived heterotypic spheroids in recapitulating tumor heterogeneity as observed in patients remains challenging given the lack of a systematic comparison with patient tissues, which are not always available for research purposes.

The integration of a microfluidic device to host the 3D CRC spheroid culture in hydrogel has enabled greater control over the cellular environment during therapeutic screening for monoculture (47–49) as well as heterocellular spheroids (50, 51), including critical molecular gradients to resemble *in vivo* conditions more closely (52). CRC spheroid models are increasing their heterogeneity by incorporating elements of the gut microbiome, a unique component of the TME of CRC, which heavily influences disease progression and response to anti-tumor therapies (53, 54). Therefore, in this mini-review, we report the recent research progress towards incorporating different cell populations in 3D CRC spheroid models, namely CAFs, tumor-associated macrophages (TAMs), ECs, and gut bacterial cells to mimic the TME heterogeneity. Differently from existing reviews, we focus on the significance of the heterogeneous cell populations during drug screening to improve the prediction of tumor response to therapy.

## 3D CRC spheroid models with fibroblasts

Fibroblasts are the major constituents of the CRC stroma and play an essential role in tumor cell invasion and progression (55, 56). CAFs are generally characterized by an increased expression of fibroblast activation protein (FAP) and smooth muscle alpha-actin (α-SMA) (57) triggered by secreted factors from surrounding cancer cells (58). CAFs secrete soluble factors, which include cytokines, chemokines, and growth factors such as interleukin 6 (IL-6), C-type lectin domain family 3 member B (CLEC3B), C-X-C motif chemokine 12 (CXCL12), and epidermal growth factor (EGF) to transform the TME to support tumor growth (59–62). Elevated serum levels of CAF-derived soluble factors stimulate signaling pathways that actively transform the TME to promote tumor metastasis and survival (63). For instance, the Wnt2 secreted from patient-derived CAFs has been shown to stimulate the Wnt signaling pathway, enhancing colon cancer cell proliferation and migration *in vitro* (64).

Recent evidence has revealed subpopulations of CAFs with different roles and prognostic significance in CRC (65, 66). Mosa et al., for instance, distinguished inflammatory-like CAFs (iCAFs) from contractile cancer-associated myofibroblasts (myCAFs) by reduced endogenous Wnt activity. Heterogeneous tumor organoids with iCAFs observed upregulated endothelial mesenchymal transition (EMT) markers, promoting tumor metastasis, whereas those with myCAFs did not (67). The heterogeneity of CAFs has been attributed to different origins and differences in secreted factors from cancer cells at each stage of tumor development (58, 65). Besides representing potential therapeutic targets within the TME, CAFs in heterotypic CRC spheroids contribute to angiogenic, invasiveness, and chemoresistance mechanisms, modulating and regulating inflammation and immunosuppression (68, 69). Therefore, CAFs heterogeneity should also be included in 3D CRC models, especially when screening for immunotherapeutic therapies.

**Figure 2:**
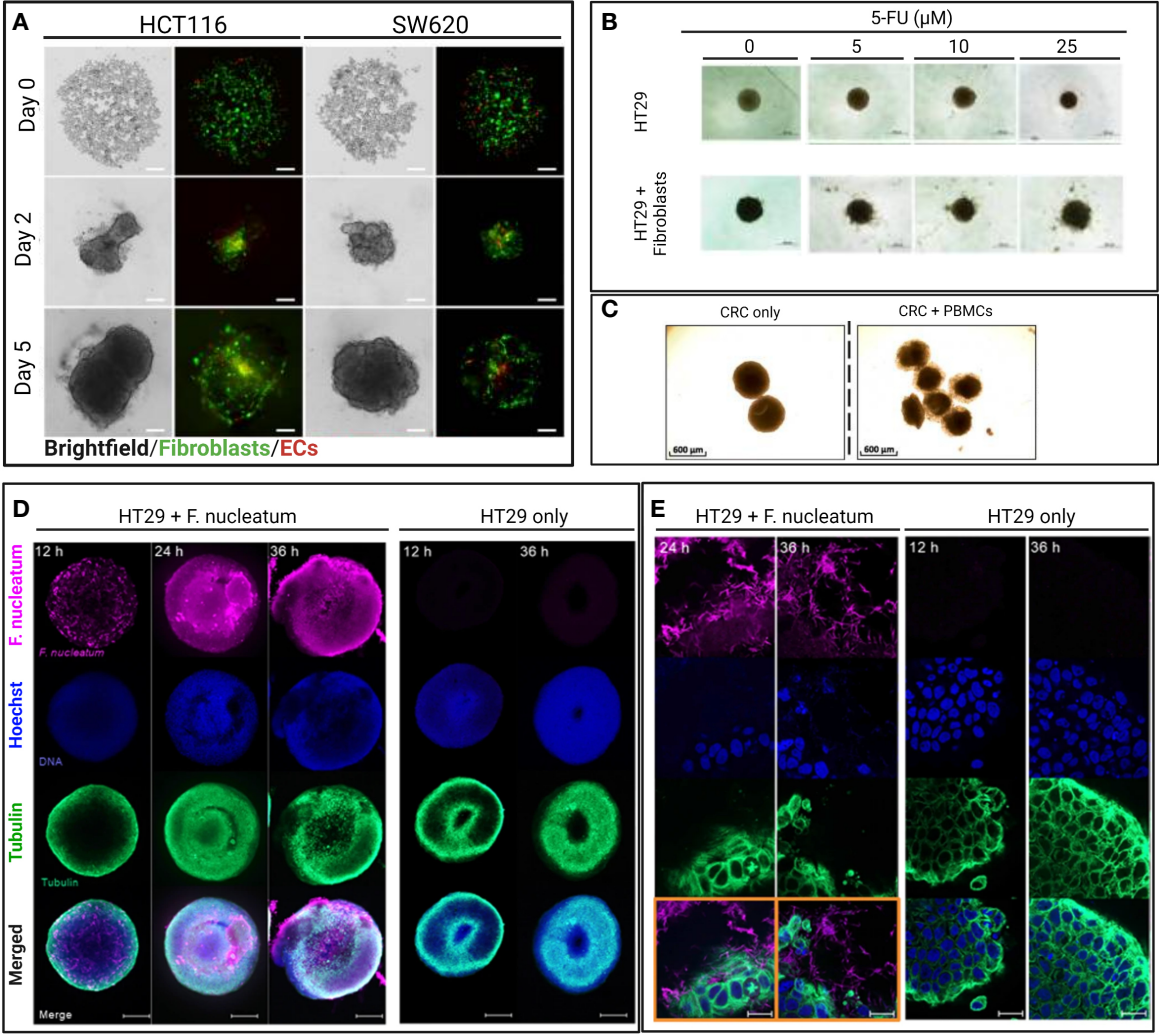
Selected images of representative heterotypic 3D CRC models from original figures of published scientific articles. **(A)** Brightfield and confocal images of intra-spheroid localization of CRC cells (HCT116 and SW620), fibroblast, and endothelial cells over time. Spheroids were formed with a 1:1 ratio of cancer cells (HCT116, SSW620) and normal human colon fibroblasts (CCD18co) with 5% of human immortalized ECs (ECRF24) and used to study drug sensitivity (45). Scale bar = 200 µm. **(B)** Brightfield images of monoculture and heterotypic HT29 spheroids treated with different concentrations of 5-FU for 48 h. Heterotypic spheroids consisted of HT29 spheroids co-cultured with (2 x 10^5^) activated MRC-5 fibroblasts (70). Scale bar = 500 µm. **(C)** Brightfield images of 3D CRC models cultured for 48 h and consisting of cancer cells (HT29) and CD19^-^CD14^-^ peripheral blood mononuclear cells (PBMC) from healthy donors to study immunomodulatory antibodies (43). Scale bar = 600 µm. **(D)** Confocal images of bacteria-spheroid co-culture consisting of HT29 cancer cells and Fusobacterium nucleatum (labeled in pink), and HT29 only spheroid at 12, 24, and 36 h. Scale bar = 200 µm. **(E)** High magnification (63 x) confocal images of 3D CRC spheroids at 12 and 36 h with and without F. nucleatum (in pink) (71). Scale bar = 20 µm.

To study the impact of fibroblasts during drug screening, Zoetemelk et al. developed a multi-cellular CRC spheroid model grown from various CRC cell lines (DLD1, HCT116, SW620) in the absence (monoculture) and presence (co-culture) of normal human fibroblasts (CCD18co) within the spheroids (45). CRC spheroids were cultured within 96 u-bottom well plates with a 0.2% gelatin-coated surface with up to 70% fibroblast population in a mixture of cell culture media (DMEM, RPMI and EMEM) supplemented with 2.5% Matrigel^®^. The co-culture spheroids also included a 5% immortalized human EC population to mimic the tumor stroma better (**Figure 2A**). Some of the co-culture spheroids displayed a higher metabolic activity and survival compared to monoculture CRC spheroids after 72 h of treatment with chemotherapeutic drugs regorafenib, erlotinib, and 5-fluorouracil (5-FU). The co-culture spheroids also displayed striking morphological differences compared to the monoculture spheroids, whereby the co-culture spheroids were characterized as irregularly shaped and with multi-directional outgrowth. The decreased sphericity potentially contributed to the enhanced survival of the co-culture spheroids by increasing the surface area for improved exchange of oxygen and nutrients. The co-culture spheroids exclusively produced fibronectin, an extracellular matrix component that assists tumor growth, progression, and invasion (72). Notably, this heterotypic 3D culture was maintained for up to 10 days, wherein the spheroids observed sustained continuous proliferation measured by their increasing diameter, making it ideal for studies on drug testing lasting up to 10 days. Zoetemelk et al. simulated a multi-dose regimen in patients through an additional 48 h treatment of their co-culture spheroids after the initial 72 h incubation, which improved the treatment efficacy in comparison to the single high-dose drug administration. Zoetemelk et al. demonstrated that the contribution of fibroblasts to tumor survival during chemotherapy was successfully recapitulated in the co-culture spheroids and the possibility for heterotypic spheroids to test multi-dose regimens *in vitro*.

Dolznig et al. generated co-culture CRC spheroids in collagen matrix containing 10x PBS, fibroblast growth medium (FGM)/20% methylcellulose and collagen in 1:4:5 volumetric ratio at neutral pH (41). They considered various colon adenocarcinoma cell lines (LS174T, HCT116, SW480, SW620, Colo205, and HT29), colon fibroblast cell lines (CCD18Co, Caco-2, and BJ-1), and CAFs isolated from patients. The tumor model containing CAFs and LS174T cells presented an enhanced invasive potential of the cancer cells and a higher percentage of the nuclear β-catenin positive cells, indicating the Wnt pathway activation as observed in patients (41). This co-culture CRC spheroid model was applied to evaluate the therapeutic efficacy of PI3K inhibitor LY294002 in FGM supplemented with 2.5% serum, observing up to a 3-fold suppression of spheroid growth over 7 days of incubation (41).

The enhanced fibroblast-associated cancer cell proliferation and migration in patients makes CAFs potential therapeutic targets. Consequently, researchers can use 3D CRC spheroid models to test therapeutic strategies modulating CAFs (alone or in combination) as done by Dana et al. (70) (**Figure 2B**). They demonstrated resveratrol-loaded liposomes (L-RES) therapeutic efficacy in reducing fibroblast activation and increasing drug sensitivity of co-culture spheroid during 5-FU treatment. The co-culture spheroids were formed with HT29 colorectal adenocarcinoma cell line and human lung fibroblasts MRC-5 cultured for 3 days in 96 well round bottom ultra-low attachment plates. For the drug sensitivity assay, the co-culture CRC spheroids were treated with 25 μM of L-RES in combination with 5-FU at concentrations of 5–25 μM for 2 more days (70).

All the described 3D CRC spheroid models well mimicked the fibroblast-associated chemoresistance and cancer progression observed in patients (64), supporting the importance of recapitulating the cell heterogeneity within the TME in heterotypic 3D CRC spheroid models for drug testing. In addition to cell viability, proliferation, and migration previously mentioned as quantifiable parameters, several metastatic biomarkers (e.g., AGR2, CacyBP, and EphA2) could be measured in these 3D models to further assess changes in the tumor metastatic potential. As observed in Zoetemelk et al. (45), CRC spheroid culture can be designed to accommodate multi-dose drug testing, although 3D *in vitro* tumor models are mostly conceived to achieve a fast prediction of the drug efficacy to speed up the drug development rather than establish long-term cultures.

## 3D CRC spheroid models with fibroblasts and immune cells

Colorectal tumors often observe a robust population of infiltrating immune cells and an increased expression of pro-inflammatory cytokines (73). Immune cells, specifically TAMs, and their secreted cytokines are essential components of the TME, significantly influencing tumor progression, immunosuppression, and, indirectly, chemoresistance (74, 75). In particular, TAMs in CRC, as in many solid tumors, consist of pro-inflammatory M1-like and anti-inflammatory M2-like macrophages, with a dynamic population ratio that varies with tumor progression (76). Väyrynen et al., for instance, observed that higher cancer survival was associated with higher density of M1-like macrophages than M2-like macrophages (77), in agreement with the evidence of M1-like macrophages having anti-tumor properties (e.g., inhibiting angiogenesis and tumor cell infiltration) whereas M2-like macrophages promoting tumor progression (78). This suggests that the roles and functions of TAMs must be considered during drug screening in 3D CRC spheroid models. However, we found only a few examples in the literature of 3D CRC spheroid models including immune cells that were applied for testing therapeutics.

To the best of our knowledge, the only 3D CRC spheroid model including both fibroblasts and macrophages within the tumor spheroid used for chemotherapeutic screening was presented by Bauleth-Ramos et al. (42). A heterogeneous CRC spheroid model was formed in 7 days, consisting of (90.8 ± 2.4%) CRC cancer cells (HCT116), (5.6 ± 1.6%) human intestinal fibroblasts, and (7.5 ± 1.2%) macrophages matured from blood derived monocytes from human donors. The spheroids were developed in agarose micro-molds produced with 3D Petri Dish® and cultured in 12 well plates with RPMI medium for the evaluation of a combined chemo-immune treatment for 48 h. The macrophages, identified by flow cytometry as mixed M1/M2 population with a major proportion of M2-like macrophages, promoted continuous tumor cell proliferation in spheroids through days 1 to 7, whereas spheroids lacking the macrophages demonstrated stagnant growth. The metastatic potential of the CRC spheroids was observed as cell dispersion from the spheroids but not quantified to compare monoculture and triculture (42). However, the consistent increase in diameter over time of the triculture CRC spheroids suggested a tumor-promoting role of M2-like macrophages in line with previous literature (79). The spheroids with increasing heterogeneity were treated with the chemotherapeutic Nutlin-3a (Nut3a) and granulocyte-macrophage colony-stimulating factor (GM-CSF) loaded in spermine-modified acetylated dextran nanoparticles (NPs). The Nut3a-loaded NPs showed a dose-dependent anti-proliferative effect in triculture and promoted the M1 over M2 polarization in spheroids as measured by the ratio of CD163 (M2 marker) to CD86 (M1 marker) expression (42).

Aside from macrophages, other immune cells are found in the TME and play important roles in regulating tumor growth, metastasis, and drug sensitivity, including dendritic cells (80), T cells, and NK cells (81). Courau et al. demonstrated that T cells and NK cells, enriched from human donor peripheral blood mononuclear cells (PBMC) and co-cultured with CRC spheroid in RPMI medium in 96 well plates after spheroid formation, successfully infiltrated the spheroids to initiate tumor cell apoptosis after 48 h (**Figure 2C**) (43). T and NK cells' contribution to the CRC TME is important, especially for screening immunotherapies that have yet to achieve satisfying clinical efficacy as CRC treatment (82). For instance, Herter et al. developed a CRC spheroid model with cancer cells (LoVo and LS174T) and fibroblasts (CCD18Co) in an FGM-2 medium to evaluate an interleukin-2 variant, IgG-IL2v, as novel immunotherapeutic. They measured the IgG-IL2v influence on the infiltration of human peripheral blood monocytes into the CRC spheroids after 72 h (40), highlighting the possibility of studying the influence of various immune cells infiltrated within 3D CRC spheroid models.

Triculture 3D CRC spheroid models with fibroblasts and TAMs may be considered more suited for evaluating immunotherapy strategies compared to 3D CRC spheroid models with only cancer cells or co-culture of cancer cells and fibroblasts. Further, as the crosstalk among cancer, stromal and immune cells modulate the release of immunosuppressive cytokines within the TME, impacting cell metabolisms, cell differentiation and functions (83–85), the stromal and immune components should be taken into consideration for a more comprehensive evaluation when testing not only immunotherapies (86) but any anti-tumor therapeutic, providing insights into drug mechanisms and influence over critical parameters in the TME.

## 3D CRC spheroid models with ECs

The secretion by tumor cells of pro-angiogenic growth factors, such as vascular endothelial growth factor (VEGF) and vascular endothelial growth factor receptor 2 (VEGFR2), promotes the development of new irregular blood vessels that supply tumors with nutrients and oxygen (87). The ECs contribute to a disordered TME, influencing tumor progression (88) and chemoresistance (89). The ECs associated with tumor angiogenesis have demonstrated phenotypic and genetic differences from normal ECs and are, at times, specifically referred to as tumor-associated endothelial cells (TECs) (90, 91). Consequently, TECs may influence the TME and the tumor sensitivity to drugs differently from normal ECs (92). Therefore, it is advisable to determine the nature of ECs (as “normal” or “tumor-associated”) when integrated into spheroid models to rationalize the contribution of incorporating the EC population in mimicking the TME. This determination could be performed by genomic profiling (91) or by comparing the relative expressions of key markers of TECs such as biglycan (93).

As anticipated, Zoetemelk et al. introduced a 5% cell population of human immortalized vascular endothelial cells, ECRF24, in their heterogeneous human 3D CRC spheroid model containing cancer cells and fibroblasts (45). The authors discussed the spatial localization of ECs close to fibroblasts in the center of the spheroids for those formed with DLD1, SW620, and HCT116 cells. However, since the drugs were screened on either monoculture or triculture condition, no specific association between the spheroid sensitivity and EC presence was possible.

More recently, Carvalho et al. published a quadruple multi-cellular human CRC spheroid model by co-culturing HCT116 with human intestinal fibroblasts (HIFs), human pulmonary microvascular endothelial cells (HPMECs) and human monocytes to mimic a pro-angiogenic TME and test anti-angiogenic nanoparticles (NPs) containing bevacizumab (BVZ) (94). Three different ratios of HCT116:HPMECs:HIFs:monocytes (1:1:1:1, 1:4:4:4 and 1:4:1:4) were tested to form spheroids on agarose micro-molds and cultured in RPMI medium over 7 days. The 1:1:1:1 model contained the highest expression of angiogenic CD31 marker and was selected to best recapitulate the pro-angiogenic TME. The NPs-based treatment resulted in the reduction of the endothelial cell marker CD31 and consequently reduced the angiogenic potential of the CRC spheroids, demonstrating the efficiency of the CRC model in screening anti-angiogenic drugs and nanoparticles. Furthermore, while not discussed, the high heterogeneity of the model by inclusion of stromal, endothelial and immune cells also enables the evaluation of chemo-immunotherapy strategies and multi-action drugs, although a different cell ratio may be optimal.

While vascularized heterotypic CRC spheroids will indeed represent a pathophysiologically relevant TME for drug screening (95) and studying the permeation of drugs through vasculature (96), few vascularized CRC models (97, 98) have been presented.

## 3D CRC spheroid models with gut bacteria

The gut microbiome is among the most important environmental factors contributing to CRC development (53, 99, 100). The gut microbiome consists of several micro-organisms, including bacteria, viruses, and fungi (54, 99, 101). Over 1000 species and 7000 strains of bacteria may be found in an adult gut (53). Disturbances to the gut microbiome balance, such as an individual’s psychosocial stress or consuming antibiotics, can contribute to CRC (53, 54, 99, 100). For example, Clostridium butyicum helps to generate butyrate, folate, and biotin, which are important for regulating epithelial proliferation, thereby mitigating the risks of specific diets for developing CRC (53). Other biotas may have the opposite effect, secreting epigenetic factors that promote CRC (102). For example, a high-fat diet can cause excessive accumulation of lipopolysaccharides, a bacteria side product, that can enter the intestinal circulation and cause inflammation which may develop into CRC (103). Apart from carcinogenesis, the gut microbiome has implications for the development of chemoresistance, and it contains potential therapeutic targets (100). For instance, Fusobacterium nucleatum (F. nucleatum) has been linked to the chemoresistance of CRC to 5-FU through two separate mechanisms (104). However, few 3D models have been developed to consider the gut microbiome’s role in CRC.

Kasper et al. developed a 3D model of a spheroid derived from CRC cell lines (HCT116 and HT29) capable of housing and promoting the growth of two strains of the anaerobic bacteria F. nucleatum (**Figures 2D, E**) after spheroid formation in McCoy’s 5 A medium (supplemented with serum) to observe bacteria-tumor cell interactions and metabolic crosstalk within the TME (71). Interestingly, the tumor-bacteria spheroids shown an enriched IL-8 metastatic signaling, mirroring the increased IL-8 expression in CRC patients with high F. nucleatum. IL-8 has been shown to promote proliferation and survival of cancer cells (105, 106). However, the model has a limited culture time because the F. nucleatum induced tumor cytotoxicity after 24 h (71). Therefore, this human tumor-bacteria co-culture in a 3D setting should be further optimized for evaluating potential drug candidates or therapeutic regimens for treating CRC while considering the potential chemoresistance induced by F. nucleatum.

Lee et al. evaluated the potential anti-cancer activity of another component of the gut microbiome, the probiotic bacterium Lactobacillus fermentum (grown and expanded in Lactobacilli De Man, Rogosa, Sharpe broth), in their 3D CRC spheroid model cultured in RPMI medium (supplemented with serum) in 96 well round bottom plate (107). The effect of Lactobacillus fermentum was observed through increased apoptosis of HCT116 cells after 72 h, which was observed solely in the 3D CRC model and not in 2D monolayer cultures (107). Rubert et al. instead demonstrated that the native (poly)phenols and gut microbial metabolites inhibited the propagation and viability of HCT116 spheroids cultured in RPMI medium (supplemented with serum) in 96 well round bottom plate after 72 h incubation (108).

Indeed, the gut microbiome’s influence on CRC progression, survival, and chemoresistance warrants research work to determine their potential as therapeutic targets. However, 2D cultures are insufficient to assess the gut microbiota's activity in CRC (107). In this resepct, 3D CRC spheroid models provide an attractive *in vitro* strategy for exploring the specific role of gut microbiota in influencing chemoresistance, tumor progression, and survival. Drug evaluations in human 3D CRC spheroid models should, therefore, include a systematic evaluation of the activity of the gut microbiota to better appreciate their role in the TME during treatment.

## Conclusions

There needs to be more standardization and validation of the methodologies for applying human 3D CRC spheroid models to preclinically assess the efficacy of drugs or other therapeutic strategies. This limitation has challenged the reproducible implementation of 3D spheroid models in drug development and confidence in the drug efficacies observed (109). Monoculture spheroids are simple and quick to optimize, justifying their use for high-throughput screening of drugs until the processes for heterogeneous spheroid formation, treatment, and assessment are better validated and automated. Indeed, heterogeneous spheroids have been demonstrated to have pathophysiological similarities and relevance to native tumor tissue. By incorporating fibroblasts, ECs, TAMs, and gut microbiota, human CRC spheroid models enable more in-depth investigations into the role of specific cell populations on tumor progression, survival, and chemoresistance unfeasible in traditional 2D cultures and spheroid monoculture. Diverse cell populations within the 3D models also represent attractive therapeutic targets that cannot be identified and validated in monoculture. Heterotypic 3D CRC spheroids thereby offer great potential for more precise predictions of the efficacy of chemotherapies to aid the discovery and development of new drug candidates, representing a promising preclinical tool for overcoming some of the limitations of previous *in vitro* and *in vivo* models.

## Author contributions

JY manuscript writing, GA review and editing. All authors have read and agreed to the published version of the manuscript. All authors contributed to the article and approved the submitted version.
